# Ubiquitous giants: a plethora of giant viruses found in Brazil and Antarctica

**DOI:** 10.1186/s12985-018-0930-x

**Published:** 2018-01-24

**Authors:** Ana Cláudia dos S. P. Andrade, Thalita S. Arantes, Rodrigo A. L. Rodrigues, Talita B. Machado, Fábio P. Dornas, Melissa F. Landell, Cinthia Furst, Luiz G. A. Borges, Lara A. L. Dutra, Gabriel Almeida, Giliane de S. Trindade, Ivan Bergier, Walter Abrahão, Iara A. Borges, Juliana R. Cortines, Danilo B. de Oliveira, Erna G. Kroon, Jônatas S. Abrahão

**Affiliations:** 1 Laboratorio de Vírus, Departamento de Microbiologia, Instituto de Ciências Biológicas, Universidade Federal de Minas Gerais, Belo Horizonte, Brazil; 2 Laboratório de Diversidade Molecular, Instituto de Ciências Biológicas e da Saúde, Universidade Federal de Alagoas, Maceió, Brazil; 3 Departamento de Patologia, Universidade Federal do Espírito Santo, Maruípe, Brazil; 4 Department of Microbiology, Icahn School of Medicine at Mount Sinai, New York, NY USA; 5 Instituto do Petróleo e dos Recursos Naturais (IPR), Pontifícia Universidade Católica do Rio Grande do Sul, Porto Alegre, RS Brazil; 6 Department of Biological and Environmental Sciences, University of Jyvaskyla, Jyvaskyla, Finland; 7 Embrapa Pantanal, Corumbá, Brazil; 8 Universidade Federal de Viçosa, Viçosa, Brazil; 9 Departamento de Virologia, Universidade Federal do Rio de Janeiro, Rio de Janeiro, Brazil; 10 Faculdade de Medicina, Universidade Federal do dos Vales do Jequitinhonha e Mucuri, Diamantina, Brazil

**Keywords:** Giant viruses, Prospection, Brazil, Antarctica, Pandoravirus, Cedratvirus, Marseillevirus, Mimivirus

## Abstract

**Background:**

Since the discovery of giant viruses infecting amoebae in 2003, many dogmas of virology have been revised and the search for these viruses has been intensified. Over the last few years, several new groups of these viruses have been discovered in various types of samples and environments.In this work, we describe the isolation of 68 giant viruses of amoeba obtained from environmental samples from Brazil and Antarctica.

**Methods:**

Isolated viruses were identified by hemacolor staining, PCR assays and electron microscopy (scanning and/or transmission).

**Results:**

A total of 64 viruses belonging to the *Mimiviridae* family were isolated (26 from lineage A, 13 from lineage B, 2 from lineage C and 23 from unidentified lineages) from different types of samples, including marine water from Antarctica, thus being the first mimiviruses isolated in this extreme environment to date. Furthermore, a marseillevirus was isolated from sewage samples along with two pandoraviruses and a cedratvirus (the third to be isolated in the world so far).

**Conclusions:**

Considering the different type of samples, we found a higher number of viral groups in sewage samples. Our results reinforce the importance of prospective studies in different environmental samples, therefore improving our comprehension about the circulation anddiversity of these viruses in nature.

**Electronic supplementary material:**

The online version of this article (doi: 10.1186/s12985-018-0930-x) contains supplementary material, which is available to authorized users.

## Background

The discovery of *Acanthamoeba polyphaga mimivirus* (APMV) in 2003, the first isolated giant virus infecting amoebas, interested the scientific community due to its size and genome content, which culminated in the search for and isolation of new giant viruses [1, 2]. The giant amoebal viruses have many phenotypic and genomic features which had never been seen in other viruses before, like large viral particles presenting up to 1.5 μm in length and large double-stranded DNA genomes ranging from 350 kb in *Marseilleviridae* members to 2500 kb for pandoravirus [3, 4]. These genes encode many hypothetical proteins, uncharacterized, or with functions that have never orrarely been observed before in other viruses, such as those related to translation and DNA repair [5–7]. Common characteristics shared by giant and large DNA viruses permitted their incorporation into a supposedly viral monophyletic group, named nucleocytoplasmic large DNA viruses (NCLDV), created in 2001 [8]. When the NCLDV group was proposed, it was composed of families *Poxviridae (*e.g. *Vaccinia virus, Crocodilepox virus)*, *Asfarviridae (*e.g. *African swine fever virus*) *Iridoviridae* (e.g. *Frog virus 3)* and *Phycodnaviridae* (e.g. *Emiliania huxleyi virus 86, Aureococcus anophagefferens virus)* [8].

Subsequently, viruses belonging to the Mimiviridae, Marseilleviridae, Ascoviridae family and also the pandoravirus, faustovirus, pithovirus, mollivirus, kaumoebavirus, cedratvirus and pacmanvirus were also incorporated to NCLDV group [9–17]. Recent prospective studies have shown that giant viruses are ubiquitous, as are their protozoa hosts [2, 18, 19]. The use of high-throughput techniques and different species of amoebae in culture for viral isolation has allowed the discovery of a large variety of new viruses and new lineages in recent years. They have been detected and/or isolated in all continents of Earth. Metagenomic studies have indicated an outstanding profile of giant virus distribution and diversity in natural environments and organisms, including water, soil, invertebrates and mammals [14, 20–27]. It is important to note that mimiviruses and marseilleviruses have also been isolated from human samples, raising questions abouttheir possible role as pathogenic agents of diseases, but this possibility still under investigation, and these viruses may be components of healthy humans virome [24, 25, 28–31].

Despite the advances made in the techniques used to isolate new giant viruses, which have increased the success of detection and the isolation of these viruses in different environments around the world, the diversity, distribution and role of these viruses in nature is still far from completely understood. Therefore, in order to better understand the diversity and distribution of giant viruses in the environment, this work aimed at the isolation and identification of giant viruses obtained from clinical and environmental samples from different regions of Brazil and Antarctica. A total of 976 samples were analyzed and 68 viruses were isolated. Taken together, our results reinforcethat giant viruses, in particular mimiviruses, are ubiquitous and may play an important role in the control of amoebal populations, both in natural and anthropogenic-affected environments.

## Methods

### Samples collection and treatment

**Fig. 1:**
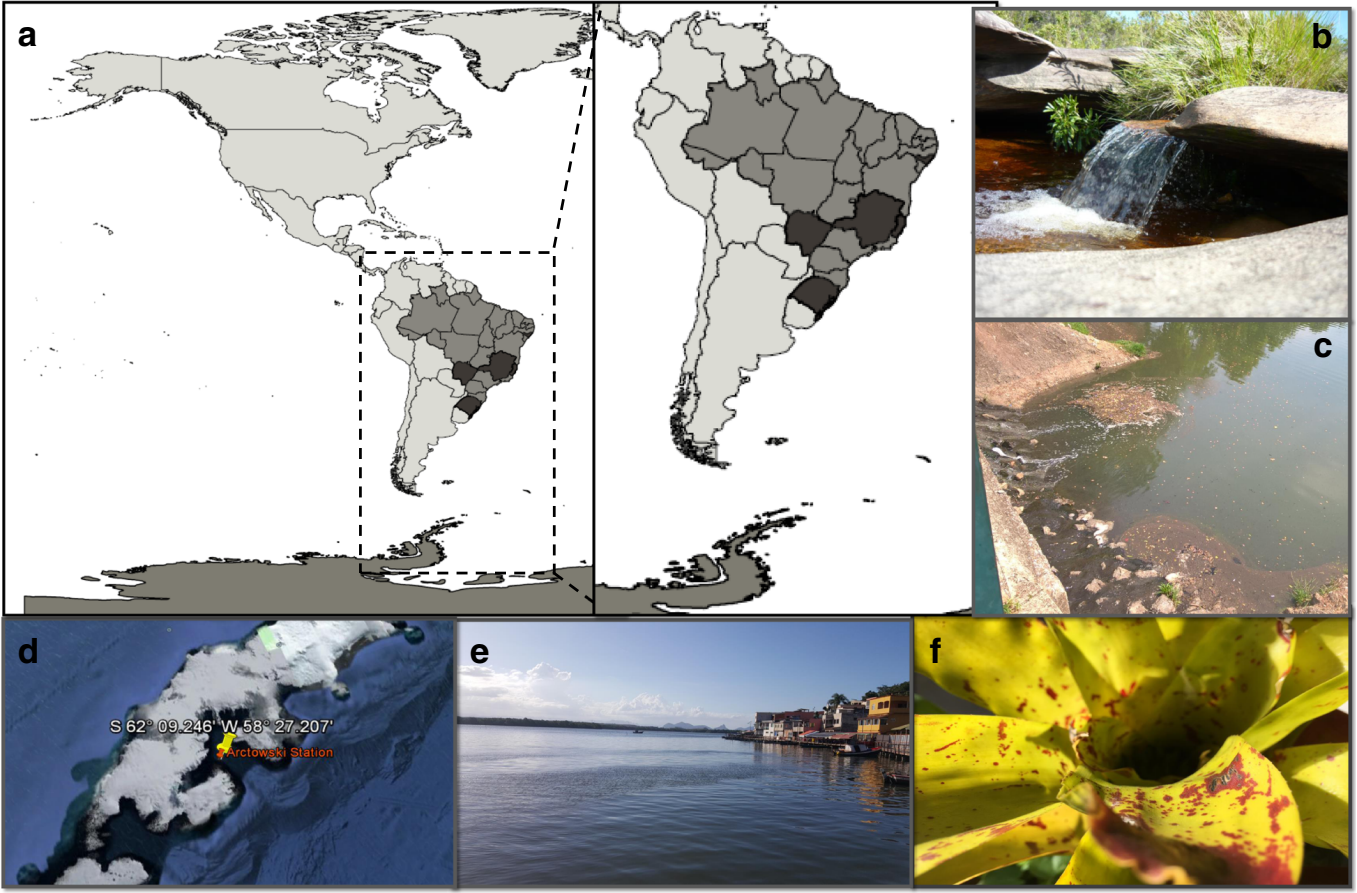
Locations where the environmental samples were collected. Schematic map (**a**) indicating in dark gray the location of collections tested and pictures from representative areas represented by letters (**b**-**f**). River at Serra do Cipó, MG, Brazil (**b**); Sewage creeks, Belo Horizonte, MG, Brazil (**c**); Location of collection of one of marine water samples in Antarctica (**d**); Location of collection of mangrove water, ES, Brazil (**e**). Bromeliads at Serra da Saudinha, AL, Brazil (**f**)

In this work, a collection composed of 976 clinical and environmental samples was analyzed: 495 soil samples (mean weight was 3 g of each sample), 124 water samples (10 mL of each sample), 140 sewage samples (10 mL of each sample), 200 human nasopharyngeal aspirate samples (1,5 mL of each sample) and 17 capybara samples (mean weight was 2 g of each sample) (Table 1 and Fig. 1). All collections were collected in different locations using sterile tubes.

**Table 1:** Collections and locations of samples analyzed

Collections	Type of sample	Collection site	Date of collection
Serra do Cipó			
13 samples	Freshwater	Serra do Cipó, MG, Brazil	Jan.2015
47 samples	Soil	Serra do Cipó, MG, Brazil	Jan. 2015
Sewage creeks Pampulha			
110 samples	Sewage	Pampulha Creeks, Belo Horizonte, MG, Brazil	Oct.2016
Farm Sewage			
30 samples	Sewage	Itaúna, MG, Brazil	Nov. 2016
Water treatment station			
50 samples	Freshwater	COPASA, Belo Horizonte, MG, Brazil	Dec. 2016
Antarctic			
7 samples	Marine Water	Antarctic	Dec. 2014
Capybara Stool			
17 samples	Stool	Serra do Cipó, MG, Brazil Pampulha, MG, Brazil Serro, MG, Brazil Pantanal, MS, Brazil	Dec. 2012 Dec. 2012 Dec. 2012
Minas Gerais Soil			
470 samples	Soil	MG, Brazil	Jan. 2014
Pantanal soil			
12 samples	Soil	Pantanal, MT, Brazil	Mar. 2015
Human nasopharyngeal aspirate			
200 samples	Human nasopharyngeal aspirate	Laboratório Central do Estado do Rio Grande do Sul, Brazil	Nov. 2014
Bromeliads Water			
10 samples	Freshwater	Maceió, AL, Brazil	Set. 2015
Mangrove water			
10 samples	Mangroove water Marine water	ES, Brazil	Feb. 2015

The samples of human nasopharyngeal aspirate were used under approval of the ethics committee of Universidade Federal de Ciências da Saúde de Porto Alegre (protocol number 1774/12, register 928/12). After collection, all samples were stored at 4 °C until inoculation procedures were performed.

Initially, the samples were divided into two groups, one with sediment-free water, including human clinical samples and other with a high concentration of sediment and soil. Samples with only water and no sediment were directly inoculated onto amoebalcultures. The soil samples were transferred to conical tubes of 15 mL and treated with 5 mL of phosphate buffered saline (PBS). The system was left for 24 h for sediment decantation and then the supernatants were collected and inoculated onto amoebal cultures.

### Culture procedures

For viral isolation, we used *Acanthamoeba polyphaga* (ATCC 30461), *Acanthamoeba castellanii* (ATCC 30234) kindly provided by the Laboratório de Amebíases (Departamento de Parasitologia, ICB/UFMG) and *Vermamoeba vermiformis* (ATCC CDC19), kindly provided by Professor Bernard La Scola from Aix Marseille University. Amoeba were grown in 75 cm^2 ^Nunc™ Cell Culture Treated Flasks with Filter Caps (Thermo Fisher Scientific, USA) with 30 mL of peptone-yeast extract-glucose (PYG) medium supplemented with 0,14 mg/mL penicillin (Sigma-Aldrich, USA), 50 mg/mL gentamycin (Thermo Fisher Scientific, USA) and 2.5 mg/mL amphotericin (Bristol-Myers- Squibb, New York, USA) at 32 °C.

For co-culture, amoeba were re-suspended in 10 mL of PYG supplemented with an antibiotic mix containing 0,004 mg/mL ciprofloxacin (Cellofarm, Brazil), 0,004 mg/mL vancomycin (Sigma-Aldrich, U.S.A), and 0,020 mg/mL doxycycline (Sigma-Aldrich, U.S.A). The suspension was then diluted 1:10 in PBS and then inoculated in96-well plates containing 4 × 10^4^ cells per well. The plates were incubated for 7 days at 32 °C and observation of the cytopathic effect was done daily using an inverted optical microscope. The well contents were then collected, frozen and thawed three times to lyse the bacterial and fungal cells that may be present in the samples and thereby decrease the chance of co-culture contamination and also helps release the viruses of amoeba cells not lysed. Posteriorly, the samples were re-inoculated for two new sub-cultures on fresh amoeba, as described above (blind passages). The contents of wells with cytopathic effect were collected and inoculated in a new 25 cm^2 ^Nunc™ Cell Culture Treated Flasks with Filter Caps (Thermo Fisher Scientific, USA) culture containing 1 million cells, the cytopathic effect was confirmed and this culture was centrifuged 10,000 rpm for 10 min (Centrifuge Sigma 1–14) for lysate clearance and were further analyzed for giant viruses. Negative controls with no sample inoculated amoeba were used in all microplates.

### DNA extraction and PCR

After the identification of cultures with a cytopathic effect, screening was done to identify which giant virus was present in samples using PCR with specific targets for some giant virus groups (Table 2). For this, 200 μL of each positive suspension was used for DNA extraction. DNA was extracted using the phenol-chloroform method [32] and used at the concentration 50 μg/μg as a template for PCR assays. The genes targeted in the PCR assays were: helicase of mimivirus lineage A; DNA polymerase B of mimivirus lineage B; DNA polymerase B of mimivirus lineage C; the major protein of the capsid of the family *Mimiviridae* (generic reaction targeting lineages A, B and C), *Marseilleviridae*, pandoravirus and cedratvirus. The primers were designed using a freely available primer design tool (https://www.ncbi.nlm.nih.gov/tools/primer-blast/) at the National Center for Biotechnology Information, U.S.A (NCBI); the sequences are described in Table 2. The primers and reactions were designed and standardized considering all analyzed viruses available on GenBank to avoid cross-amplification. PCR assays were performed using 1 μL of extracted DNA (~ 50 nanograms) in an amplification reaction mix containing 5 μL of SYBR Green Master Mix and 0.4 μL (10 μM) of forward and reverse primers. The final volume of the reaction was adjusted with ultrapure waterto 10 μL. The conditions of the StepOne thermal cycler reactions (Applied Biosystem, USA) were: 95 °C for 10 min, followed by 40 cycles of 95 °C for 15 s and 60 °C for 1 min, which was followed by a final step of 95 °C for 15 s, 60 °C for 1 min and 95 °C for 15 s. Positive samples in the PCR were those that amplified, showing the specific melting temperature, using the primers listed in Table 2, whereas the negative samples did not amplify in the PCR. As negative controls we used DNA extracted from non-inoculated amoebas with purified viruses or samples, and as a positive control we used DNA from amoebae infected with purified virus. Samples that were not possible to identify using the PCR assay were identified by electron microscopy and/or hemacolor staining.

**Table 2:** Primer sequences used for specific PCR

Target genes	Forward sequence	Reverse sequence
Helicase of mimivirus lineage A	5’-ACCTGATCCACATCCCATAACTAAA-3′	5’-GGCCTCATCAACAAATGGTTTCT-3′
DNA polymerase beta of mimivirus lineage B	5’-AGTTCATCCGCACTTGGAGA-3′	5’-TCAACGGATAAAATCCCTGGTACT-3′
DNA polymerase beta of mimivirus lineage C	5′- TCCGAATTCTATGAGGGAGAGA-3′	5’-TGTTCCTTTTTGGGAGAACCA-3′
Main protein of the capsid of the family *Mimiviridae*	5’-ACTTTATTATCATTATCAGCGAATA-3’	5’-GCTCTTAACCCTGAAGAACA-3’
Main protein of the capsid of the family *Marseilleviridae*	5’-CTTTTGCACCTGCTTCATGA-3’	5’-GCGGTAACCCTCCCACTTAT-3’
Main protein of the capsid of pandoravirus	5’-GGATGGCTCGACGTCTCTT-3’	5’-CCTYGGTRAGCAMAGGCAAC-3’
Main protein of the capsid of cedratvirus	5′- AGAGTATGCTCGCAACCACC-3’	5’-CACGTTAAGGCCGGGGTAAT −3’

### Sequencing validation and phylogeny

Four isolates were selected for sequencing validation. The genome of two pandoraviruses, the cedratvirus and one mimiviruses of lineage B positive samples were sequenced using the Illumina MiSeq instrument (Illumina Inc., San Diego, CA, USA) with the paired-end application. The sequenced reads were imported to CLC_Bio software and assembled into contigs by the de novo method. The prediction of open reading frame (ORF) sequences was carried out using the Fgenes V tool. ORFs smaller than 100aa were excluded from the annotation. Paralogous groups of genes were predicted by OrthoMCL program. The ORFs were functionally annotated using similarity analyses with sequences in the NCBI data base using BLAST tools. One fragment of 327 amino acid of DNA polymerase B gene sequence of the samples was aligned with sequences from other giant viruses, previously deposited in GenBank, using the ClustalW program. After the alignment analysis, phylogeny reconstruction was performed using the Neighbor-joining method implemented by the MEGA7 software.

### Viral stock production and titration

For seed pool production, *A. castellanii* or *A. polyphaga* cells were cultivated and infected with 500 μL of isolates. After observation of a cytopathic effect, the titer was obtained by end-point method [33]. Stocks were kept at − 80 °C freezers.

### Hemacolor staining

*A. castellanii* and *A. polyphaga* cells were infected with isolates at a M.O.I of 0.01 following the procedures described above. After approximately 18 h, amoeba became rounded, so 10 μL of the previously inoculated suspension was spread on a histological slide and fixed with methanol. The virus factories and viral particles were observed after hemacolor (Renylab, Brazil) or crystal violet (Labsynth, Brazil) staining, respectively. After, slides were analyzed under an optical microscope (OlympusBX41, Japan) with 1000X zoom.

### Electron microscopy

For transmission electron microscopy (TEM), *A. castellanii* and *A. polyphaga* cells were cultivated until the observation of 80–90% confluence and infected with the isolates in an M.O.I of 0.01. The samples were prepared as described previously [34]. Briefly, 12 h post-infection, when approximately 50% of the cells were presenting a cytopathic effect, the medium was discarded and the monolayer gently washed twice with 0.1 M phosphate buffer. Glutaraldehyde 2.5% (*v*/v) was added to the system, followed by incubation for 1 h at room temperature for fixation. The cells were then collected, centrifuged at 3000 rpm for 10 min, the medium discarded and the cells stored at 4 °C in phosphate buffer until electron microscopy analyses.

For the scanning electron microscopy (SEM) assay, the isolates were prepared onto round glass blades covered by poly-L-lysine and fixed with glutaraldehyde 2.5% in 0.1 M cacodylate buffer for 1 h at room temperature. Samples were then washed three times with 0.1 M cacodylate buffer and post-fixed with 1.0% osmium tetroxide for 1 h at room temperature. After a second fixation, the samples were washed three times with 0.1 M cacodylate buffer and immersed in 0.1% tannic acid for 20 min. Samples were then washed in cacodylate buffer and dehydrated by serial passages in ethanol solutions with concentrations ranging from 35% to 100%. They were dried at the critical CO_2_ point, transferred into stubs and metalized with a 5 nm gold layer. The analyses were completed with scanning electronic microscopy (FEG Quanta 200 FEI) at the Center of Microscopy of UFMG, Brazil.

## Results

Here, we report the screening of 976 environmental and clinical samples collected between 2014 and 2017 and the isolation of 68 giant viruses (6.97% isolation rate). Among all of the isolated viruses, 17 (25%) were isolated in *A. polyphaga* and 51 were isolated in *A. castellanii* (75%). No virus was isolated in *V. vermiformes* (Additional file 1: Table S1).

**Fig. 2:**
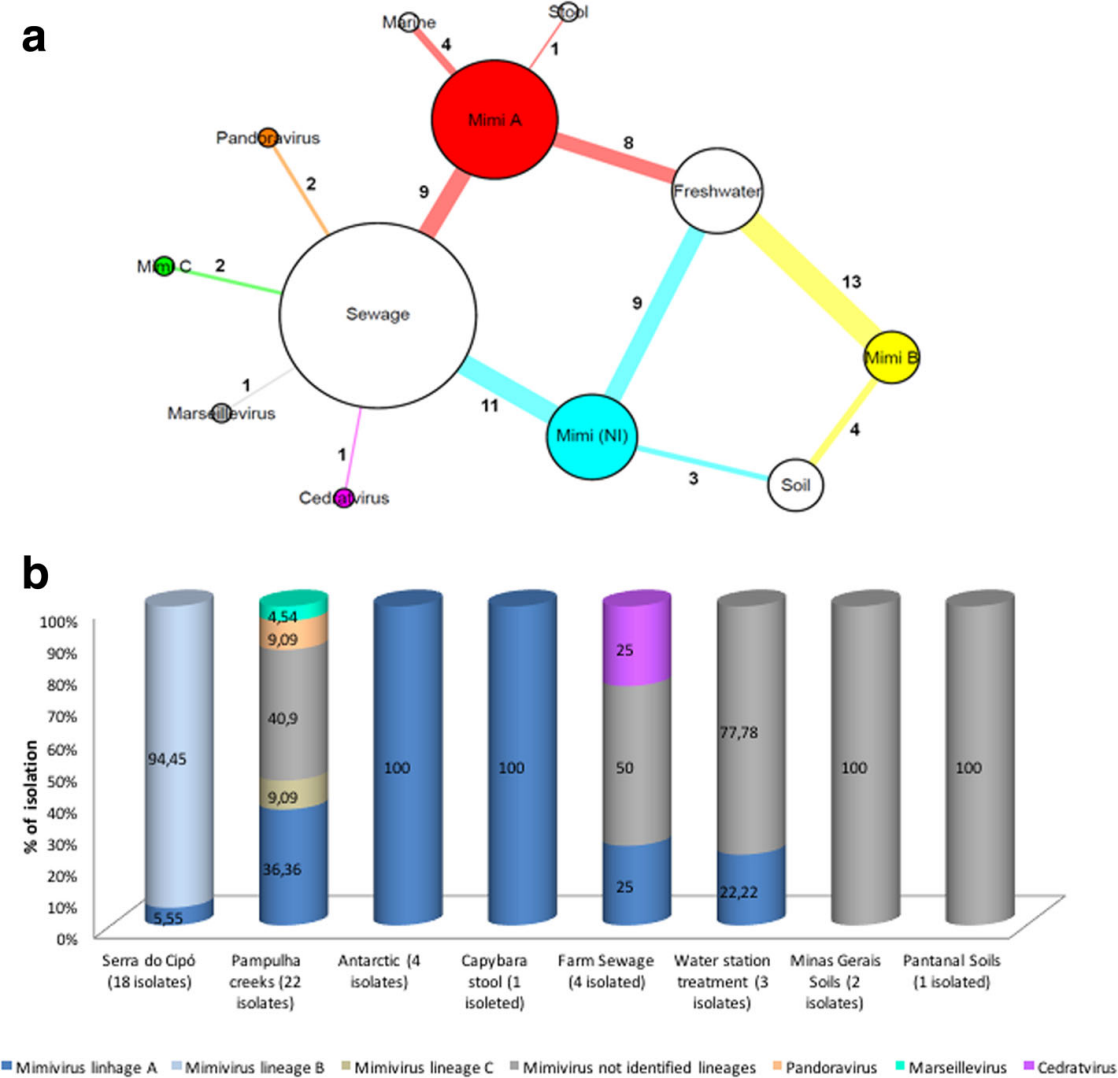
Diversity of isolated giant virus by type of sample and collections. Network graph showing the viral groups isolated and identified by PCR and electron microscopy assays in different samples. Each node represents a type of sample (white nodes) or viral group (colored nodes). The node diameter is proportional to the edge degree. The numbers of isolated viruses in each sample are shown on the respective edge. The layout was generated using a force based algorithm followed by manual rearrangement for a better visualization of the connections (**a**). A total of 7 viral groups are represented. Isolation rate of each virus groups by collections (**b**)

The PCR, hemacolor staining and electron microscopy assays showed that 22 samples were positive for mimivirus lineage A, 17 were positive for mimivirus lineage B and 2 were positive for mimivirus lineage C. In addition, 2 samples were positive for pandoravirus, 1 for cedratvirus and 1 for marseillevirus (Fig. 2). Twenty-three other samples were identified as mimiviruses by PCR (capsid gene, generic reaction), by hemacolor staining or by electron microscopy, but it was not possible to discriminate the lineage of these viruses using the specific PCR (Additional file 1: Table S1).

**Fig. 3:**
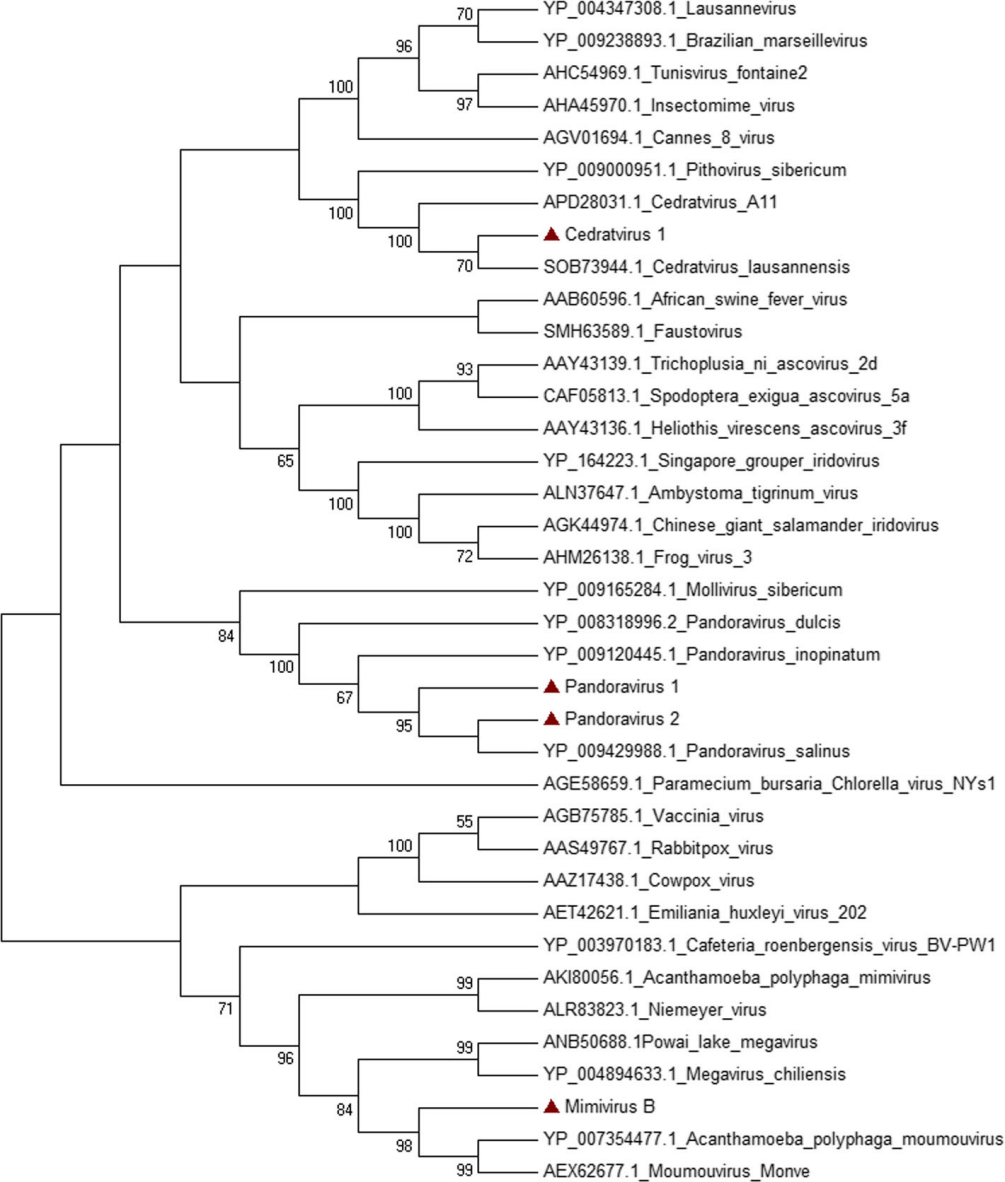
Phylogenetic tree of isolates. A neighbor-joining phylogenetic tree constructed using a 327 amino acid fragment of the DNA polymerase B gene. Tree was constructed by using MEGA version 7.0 (www.megasoftware.net) on the basis of the amino acids sequences with 1000 bootstrap replicates. Bootstrap values > 40% are shown. Nucleotide sequences were obtained from GenBank. The isolates are highlighted with red triangle. Scale bar indicates rate of evolution

Twelve samples were positive in PCR for mimivirus lineage B and for marseillevirus. Four isolates were selected for genome sequencing and phylogenetic analyzes performed with the DNA polymerase gene of these viruses confirmed the identification by PCR (Fig. 3). In order to investigate the occurrence of co-infections, these samples were analyzed via hemacolor staining, and two of them were randomly selected for diagnosis via TEM. The samples tested showed only particles with morphology similar to the mimivirus, showing no marseillevirus-like particles (Additional file 1: Table S1). In addition, no marseillevirus-like factories were observed by hemacolor staining, just mimiviruses-like ones.

The highest isolation percentages (27.42%) were obtained from the water samples, with 34 isolates from 124 samples. Of these, 4 were isolated from 7 seawater samples (57.14% isolation rate) and 30 were isolated from 117 freshwater samples (isolation rate of 25.64%) (Fig. 2).

In addition, with an isolation success of 18.57%, 26 viruses were obtained from 140 sewage samples, followed by samples of capybara feces (5.88%), with an isolate obtained from 17 samples, and soil samples (1.41%), with 7 isolates from 495 samples. In addition, 200 samples of human nasopharyngeal aspirate were tested and no isolates were obtained from these samples (Fig. 2).

Although water samples have shown the highest number of isolated virus, sewage samples presented the highest diversity of viruses groups isolated (Fig. 2). In the fresh and marine water samples, only *Mimiviridae* family viruses (12 of lineage A, 13 of lineage B and 9 unidentified) were identified, while besides *Mimiviridae* (9 of lineage A, 2 of lineage C and 11 unidentified), 1 marseillevirus, 1 cedratvirus and 2 pandoraviruses (Fig. 2) were found in the sewage samples. Soil and stool samples also showed only viruses of *Mimiviridae* family (4 of lineage B and 3 unidentified in soil, and 1 of lineage A in stool) (Fig. 2).

Comparing the percentage of isolates per collection region, we can observe that the isolation was higher in the Antarctica collection with a 57% isolation success rate; however, this collection has few samples (4 isolates from 7 samples). The collections of Serra do Cipó and sewage creeks appear with 30% (18 isolates from 60 samples) and 20% (22 isolates from 110 samples) positivity, respectively. The collections of farm sewage and Pantanal soils presented a percentage of isolations of 13.33% (4 isolates from 30 samples) and 8.33% (1 isolate from 12 samples), respectively (Fig. 2).

The collections of water station treatment, capybara stool and Minas Gerais soils, showed percentages of isolation of 8% (4 isolates from 50 samples), 5.88% (1 isolate from 17 samples) and 0,4% (2 isolates from 470 samples), respectively. Collections from bromeliad, mangrove, and human nasopharyngeal aspirate, showed no viral isolates (Fig. 2). The collection of creek sewages showed the greatest viral diversity, with isolates of *Mimiviridae*, *Marseilleviridae* and *Pandoravirus* groups; followed by the collection of farm sewage, with isolates of mimivirus and cedratvirus. In the remaining collection, only mimiviruses were identified (Fig. 2).

**Fig. 4:**
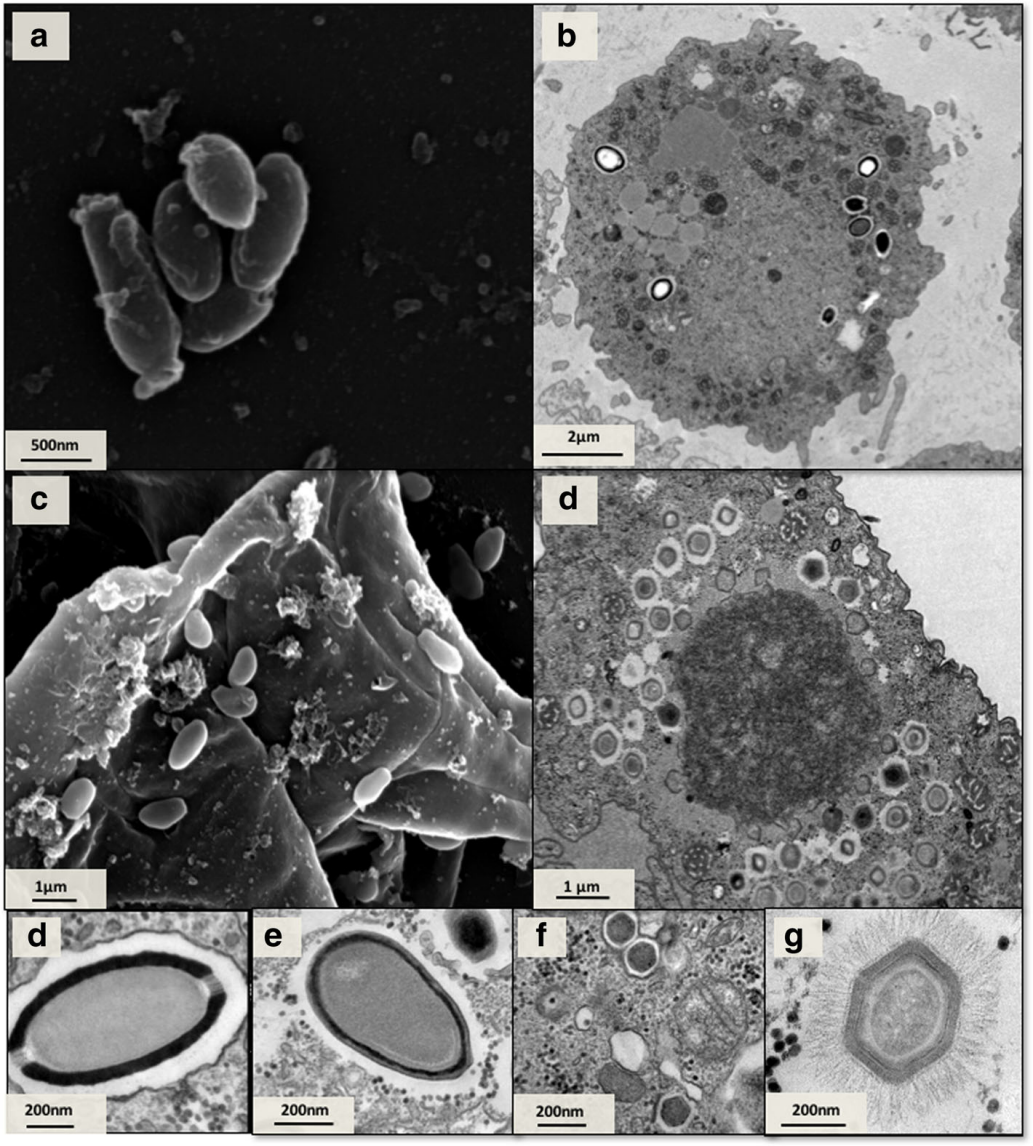
Electron microscopy images of viruses isolated. SEM of *Cedratvirus* isolated from sewage farm of MG (**a**) TEM (**b**) and SEM (**c**) of *Pandoravirus* isolate from Mergulhão sewage creek. TEM of *Mimivirus* isolated from Antarctica (**c**) TEM of *Cedratvirus* isolated from sewage farm of MG (**d**) TEM of *Pandoravirus* isolate from Bom Jesus sewage creek (**e**) marseillevirus isolated from Bom Jesus sewage creek (**f**). TEM mimivirus particle detail that was isolated from Antarctica (**g**). Scale Bars: (**a**-**d**) 500 nm; (**e**) 50 nm

Electron microscopy assays showed that two isolated samples of Mergulhão (Fig. 4b-c) and Bom Jesus (Fig. 4e) sewage creek show pandoravirus-like morphology, with particles having an average length of 1 μm, as described by Philippe and colleagues in 2013. Antarctica isolates showed a mimivirus-like morphology, with particles of about 750 nm, as described by La Scola and colleagues in 2003 (Fig. 4d and g). SEM analyses of a sample of sewage farm collection showed cedratvirus-like morphology with particles of approximately 1.2 μm, as described by Andreani and colleagues in 2016 (Fig. 4a and d). The images obtained from another isolate from Bom Jesus creek showed particles with marseillevirus-like morphology, apparently with icosahedral symmetry and dimensions of about 200 nm (Fig. 4f).

## Discussion

The search for giant viruses in environmental and clinical samples from different regions of Brazil and Antarctica resulted in 68 isolates, reinforcing the results obtained in other prospective studies involving environmental Brazilian samples, in which a large variety of giant viruses, specially mimiviruses, were isolated [18, 35–39]. The present work corroborates those studies, since 64 out of 68 viruses isolated (94.11%) were identified as mimiviruses; however, for the first time many mimiviruses of lineages B and C were isolated in Brazil.

Although Brazil is one of the most exploited countries regarding the presence of giant viruses, only two viruses of the *Marseilleviridae* family had been isolated in this territory to date. Brazilian marseillevirus and Golden marseillevirus presented a high genomic diversity, thus suggesting that these isolates form two new lineages within the family [37, 38]. This study presents the third marseillevirus isolated from Brazil. The genomic characterization of this isolate (in progress) may expand even more the plethora of marseillevirus lineages.

Regarding pandoraviruses, since their discovery in 2013, only 4 isolates have been described worldwide [18, 40–42]. These viruses form a new group among the NLCDVs, known as new TRUC (an acronym for Things Resisting Uncompleted Classification) members [10]. Here, we add two members to this club, providing the possibility of a wider study of this virus biology. In addition, this study reports for the first time the isolation of a giant virus from capybara feces. This type of sample had not yet been explored for the presence of giant viruses although DNA from poxvirus, another member of the NCLDV group, was previously detected in this collection [43].

The isolation and detection rates of giant viruses vary in the different studied samples, with noisolation in human nasopharyngeal aspirate samples and higher rates in water and sewage, followed by stool and soil samples. These results corroborate other studies in which giant viruses are more abundant in water and sewage than in soil samples [18, 19, 34, 39]. Furthermore, we demonstrated that giant viruses are not commonly found in nasopharyngeal clinical samples, as reported elsewhere [44–46]. Considering the amoebas used in this study, *A. castellanii* was shown to be more effective in the isolation of a greater diversity of giant viruses, as demonstrated by Dornas and colleagues in 2015.

The difficulty in amplifying some preserved lineage-specific regions by PCR can be explained by the high genetic diversity among these viruses [36]. This may be one of the reasons why we could not identify the strains of all mimiviruses isolated in this work. It is also important to consider that among these, there may be new strains which have not yet been described. In addition, two samples that were PCR positive for mimivirus lineage B and marseillevirus revealed only mimiviruses particles or factories when analyzed by electron microscopy or hemacolor staining. This finding also reinforces the importance ofusing a set of techniques for the identification of giant viruses, as performedin this study.

Metagenomic studies have indicated that the presence of the giant virus gene marker is common in all continents including Antarctica, a region with extreme environmental conditions [20, 21, 47, 48]. Virophages have already been isolated from this region, which is an additional indicative of the presence of giant viruses [22, 49]. However, to our knowledge, there has been no description of mimiviruses isolated in this continent to date. Nevertheless, we report the first giant amoebal viruses in Antarctica, confirming some previous expectations and the ubiquity of these microorganisms.

Altogether, our results lead us one step further into knowledge about the giant virus diversity and ecology, but important questions were raised. What could be the role of giant viruses in an extreme environment such as Antarctica? Will the host spectrum of these viruses be the same, or are they capable of infecting other more well-adapted hosts at extreme conditions? In-depth investigations regarding genetic and biological aspects of these isolates might provide some answers. Moreover, new prospecting studies, exploring different isolation strategies in environments that have never been explored around the globe, will bring insights about the ecology of giant viruses and completely new NCLDV members could be brought to light, boosting our knowledge about the diversity of this complex group within the virosphere.

## Conclusions

This work presented the isolation of different giant virus species from the prospecting study of a large collection of environmental samples, providing the isolation of viruses never previously isolated in Brazil and Antarctica. The findings of this study reinforce the idea that giant viruses are ubiquitous and open the door to further study of the biology of these isolates, which contributes to an understanding of the diversity of these viruses.

## Additional file

Additional file 1: Table S1. Identification and locations of viruses isolated. (DOCX 34 kb)

## Abbreviations

AL: Alagoas
APMV: *Acanthamoeba polyphaga mimivirus*
ES: Espírito Santo
LACEN/RS: Laboratório Central do Estado do Rio Grande do Sul
M.O.I: Multiplicity of infection
MG: Minas Gerais
NCBI: National Center for Biotechnology Information
NCLDV: Nucleocytoplasmic large DNA viruses
PBS: Phosphate buffered saline
PCR: Polymerase chain reaction
SEM: Scanning electron microscopy
TEM: Transmission electron microscopy
