# Supplementary material for: Ubiquitous giants: a plethora of giant viruses found in Brazil and Antarctica
Source: Virol J. 2018 Jan 24;15:22. doi: 10.1186/s12985-018-0930-x (PMC5784613; doi:10.1186/s12985-018-0930-x)
Supplement: Additional file 1: Table S1. — Identification and locations of viruses isolated. (DOCX 34 kb) [file 12985_2018_930_MOESM1_ESM.docx]

| Collections  **Additional file 1: Table S1: Identification and locations of viruses isolated.** |  | Passage  of  isolation | Real Time PCR | | | | | | | | Hemacolor  Staining | TEM | SEM | Amoebas  of isolation | Collection site | Coordinates | Date colection |
| --- | --- | --- | --- | --- | --- | --- | --- | --- | --- | --- | --- | --- | --- | --- | --- | --- | --- |
| Serra do Cipó | Sample |  | Mimiviridae | | | | Pandoravirus | Marseillevirus | | Cedratvirus |  |  |  |  |  |  |  |
|  |  |  | Lineage  A | Llineage  B | Lineage  C | All  lineages | Major  Capsid  Protein | Major  Capsid  Protein | Major  Capsid  Protein | |  |  |  |  |  |  |  |
| 60 | water | 2° | neg | pos | neg | neg | neg | neg | ND | | ND | mimivirus-like | ND | *A. polyphaga (ATCC 30461)* | Serra do Cipó,  MG,Brazil | S 19° 20.840'  W 43° 36.5911' | 2015 |
| 46 | water | 2° | neg | pos | neg | neg | neg | neg | ND | | ND | mimivirus-like | ND | *A. polyphaga (ATCC 30461)* | Serra do Cipó,  MG,Brazil | S 19° 20.893'  W 43° 36.555' | 2015 |
| 1 | soil | 3° | neg | pos | neg | neg | neg | neg | ND | | ND | mimivirus-like | ND | *A. polyphaga (ATCC 30461)* | Serra do Cipó,  MG,Brazil | S 19° 20.893'  W 43° 36.555' | 2015 |
| 40 | water | 3° | neg | pos | neg | neg | neg | neg | ND | | ND | mimivirus-like | ND | *A. polyphaga (ATCC 30461)* | Serra do Cipó,  MG,Brazil | S 19° 20.756'  W 43° 37.074' | 2015 |
| 5 | water | 2° | neg | pos | neg | pos | neg | neg | ND | | ND | mimivirus-like | ND | *A. polyphaga (ATCC 30461)* | Serra do Cipó,  MG,Brazil | S 19° 22.595'  W 43° 35.731' | 2015 |
| 59 | water | 3° | neg | pos | neg | neg | neg | pos | ND | | ND | mimivirus-like | ND | *A. polyphaga (ATCC 30461)* | Serra do Cipó,  MG,Brazil | S 19° 22.595'  W 43° 35.731' | 2015 |
| 47 | water | 3° | neg | pos | neg | neg | neg | pos | ND | | ND | mimivirus-like | ND | *A. polyphaga (ATCC 30461)* | Serra do Cipó,  MG,Brazil | S 19° 22.595'  W 43° 35.731' | 2015 |
| 12 | water | 3° | neg | pos | neg | neg | neg | pos | ND | | mimivirus-like | ND | ND | *A. polyphaga (ATCC 30461)* | Serra do Cipó,  MG,Brazil | S 19° 22.595'  W 43° 35.731' | 2015 |
| 3 | water | 3° | neg | pos | neg | neg | neg | pos | ND | | mimivirus-like | ND | ND | *A. polyphaga (ATCC 30461)* | Serra do Cipó,  MG,Brazil | S 19° 20.893'  W 43° 36.555' | 2015 |
| 22 | water | 3° | neg | pos | neg | neg | neg | pos | ND | | mimivirus-like | ND | ND | *A. polyphaga (ATCC 30461)* | Serra do Cipó,  MG,Brazil | S 19° 22.595'  W 43° 35.731' | 2015 |
| 32 | water | 3° | neg | pos | neg | neg | neg | pos | ND | | mimivirus-like | ND | ND | *A. polyphaga (ATCC 30461)* | Serra do Cipó,  MG,Brazil | S 19° 20.756'  W 43° 37.014' | 2015 |
| 45 | soil | 3° | neg | pos | neg | neg | neg | pos | ND | | mimivirus-like | ND | ND | *A. polyphaga (ATCC 30461)* | Serra do Cipó,  MG,Brazil | S 19° 20.756'  W 43° 37.014' | 2015 |
| 25 | soil | 3° | neg | pos | neg | neg | neg | pos | ND | | mimivirus-like | ND | ND | *A. polyphaga (ATCC 30461)* | Serra do Cipó,  MG,Brazil | S 19° 20.756'  W 43° 37.014' | 2015 |
| 57 | soil | 3° | neg | pos | neg | neg | neg | pos | ND | | mimivirus-like | ND | ND | *A. polyphaga (ATCC 30461)* | Serra do Cipó,  MG,Brazil | S 19° 20.893'  W 43° 36.555' | 2015 |
| 29 | water | 3° | neg | pos | neg | neg | neg | pos | ND | | mimivirus-like | ND | ND | *A. polyphaga (ATCC 30461)* | Serra do Cipó,  MG,Brazil | S 19° 22.595'  W 43° 35.731' | 2015 |
| 11 | water | 3° | neg | pos | neg | neg | neg | pos | ND | | mimivirus-like | ND | ND | *A. polyphaga (ATCC 30461)* | Serra do Cipó,  MG,Brazil | S 19° 20.893'  W 43° 36.555' | 2015 |
| 48 | water | 3° | neg | pos | neg | neg | neg | pos | ND | | mimivirus-like | ND | ND | *A. polyphaga (ATCC 30461)* | Serra do Cipó,  MG,Brazil | S 19° 20.893'  W 43° 36.555' | 2015 |
| 44 | water | 3° | pos | ND | ND | pos | neg | neg | ND | | ND | ND | ND | *A. polyphaga (ATCC 30461)* | Serra do Cipó,  MG,Brazil | S 19° 20.893'  W 43° 36.555' | 2015 |
| Sewage  creeks |  |  |  |  |  |  |  |  |  | |  |  |  |  |  |  |  |
| 4.5 | Sewage | 1° | pos | ND | ND | pos | neg | neg | ND | | ND | ND | ND | *A. castellanii (ATCC30234)* | Sarandi creek,  Belo Horizonte,  MG, Brazil | S 19° 86.084'  W 43° 99727' | 2016 |
| 4.2 | Sewage | 2° | neg | neg | neg | neg | neg | neg | ND | | ND | ND | mimivirus-like | *A. castellanii (ATCC30234)* | Sarandi creek,  Belo Horizonte,  MG, Brazil | S 19° 86.084'  W 43° 99727' | 2016 |
| 5.9 | Sewage | 1° | neg | neg | neg | neg | neg | neg | ND | | ND | ND | mimivirus-like | *A. castellanii (ATCC30234)* | Tijuco creek,  Belo Horizonte  ,MG, Brazil | S 19° 85978'  W 43° 98141' | 2016 |
| 8.5 | Sewage | 1° | neg | ND | ND | neg | neg | neg | ND | | ND | ND | pandoravirus-like | *A. castellanii (ATCC30234)* | Mergulhão creek,  Belo Horizonte,  MG, Brazil | S 19°86.478'  W 43° 97618' | 2016 |
| 9.2 | Sewage | 2° | neg | ND | ND | neg | pos | neg | ND | | ND | ND | pandoravirus-like | *A. castellanii (ATCC30234)* | Bom Jesus creek,  Belo Horizonte,  MG, Brazil | S 19° 86.084'  W 43° 99727' | 2016 |
| 9.10 | Sewage | 2° | neg | ND | ND | neg | neg | neg | ND | | ND | ND | marseillevirus-lke | *A. castellanii (ATCC30234)* | Bom Jesus creek,  Belo Horizonte,  MG, Brazil | S 19°86084'  W 43° 99727' | 2016 |
| 6.10 | Sewage | 2° | pos | ND | ND | pos | neg | neg | ND | | ND | ND | ND | *A. castellanii (ATCC30234)* | Tijuco creek,  Belo Horizonte  MG, Brazil | S 19° 85978'  W 43° 98141' | 2016 |
| 1.7 | Sewage | 2° | pos | ND | ND | pos | neg | neg | ND | | ND | ND | ND | *A. castellanii (ATCC30234)* | Sewage treatment station,  Belo Horizonte,  MG, Brazil | S 19° 86.084'  W 43° 99723' | 2016 |
| 2.9 | Sewage | 2° | pos | ND | ND | neg | neg | neg | ND | | ND | ND | ND | *A. castellanii (ATCC30234)* | Conflux of Sarandi and  Ressaca creek,  Belo Horizonte,  MG, Brazil | S 19° 86.084'  W 43° 99727' | 2016 |
| 8.7 | Sewage | 2° | pos | ND | ND | neg | neg | neg | ND | | ND | ND | ND | *A. castellanii (ATCC30234)* | Mergulhão creek,  Belo Horizonte,  MG, Brazil | S 19°86.478'  W 43° 97618' | 2016 |
| 17.1 | Sewage | 3° | neg | neg | pos | pos | neg | neg | ND | | ND | ND | ND | *A. castellanii (ATCC30234)* | Bom Jesus creek,  Belo Horizonte,  MG, Brazil | S 19°86084'  W 43° 99727' | 2017 |
| 17.2 | Sewage | 3° | neg | neg | neg | pos | neg | neg | ND | | ND | ND | ND | *A. castellanii (ATCC30234)* | Bom Jesus creek,  Belo Horizonte,  MG, Brazil | S 19°86084'  W 43° 99727' | 2017 |
| 17.5 | Sewage | 3° | neg | neg | neg | pos | neg | neg | ND | | mimivirus-like | ND | ND | *A. castellanii (ATCC30234)* | Bom Jesus creek,  Belo Horizonte,  MG, Brazil | S 19°86084'  W 43° 99727' | 2017 |
| 17.9 | Sewage | 3° | neg | neg | neg | neg | neg | neg | ND | | mimivirus-like | ND | mimivirus-like | *A. castellanii (ATCC30234)* | Bom Jesus creek,  Belo Horizonte,  MG, Brazil | S 19°86084'  W 43° 99727' | 2017 |
| 17b.1 | Sewage | 2° | neg | neg | neg | neg | neg | neg | ND | | mimivirus-like | ND | mimivirus-like | *A. castellanii (ATCC30234)* | Bom Jesus creek,  Belo Horizonte,  MG, Brazil | S 19°86084'  W 43° 99727' | 2017 |
| 17b.2 | Sewage | 3° | neg | neg | neg | pos | neg | neg | ND | | mimivirus-like | ND | ND | *A. castellanii (ATCC30234)* | Bom Jesus creek,  Belo Horizonte,  MG, Brazil | S 19°86084'  W 43° 99727' | 2017 |
| 17b.4 | Sewage | 3° | pos | ND | ND | pos | neg | neg | ND | | ND | ND | ND | *A. castellanii (ATCC30234)* | Bom Jesus creek,  Belo Horizonte,  MG, Brazil | S 19°86084'  W 43° 99727' | 2017 |
| 17b.6 | Sewage | 3° | pos | ND | ND | neg | neg | neg | ND | | mimivirus-like | ND | ND | *A. castellanii (ATCC30234)* | Bom Jesus creek,  Belo Horizonte,  MG, Brazil | S 19°86084'  W 43° 99727' | 2017 |
| 17b.7 | Sewage | 2° | neg | neg | neg | neg | neg | neg | ND | | mimivirus-like | ND | mimivirus-like | *A. castellanii (ATCC30234)* | Bom Jesus creek,  Belo Horizonte,  MG, Brazil | S 19°86084'  W 43° 99727' | 2017 |
| 17b.8 | Sewage | 3° | neg | neg | neg | pos | neg | neg | ND | | mimivirus-like | ND | ND | *A. castellanii (ATCC30234)* | Bom Jesus creek,  Belo Horizonte,  MG, Brazil | S 19°86084'  W 43° 99727' | 2017 |
| 17b.9 | Sewage | 3° | neg | neg | neg | pos | neg | neg | ND | | mimivirus-like | ND | mimivirus-like | *A. castellanii (ATCC30234)* | Bom Jesus creek,  Belo Horizonte,  MG, Brazil | S 19°86084'  W 43° 99727' | 2017 |
| 17b.10 | Sewage | 2° | neg | neg | pos | neg | neg | neg | ND | | mimivirus-like | ND | ND | *A. castellanii (ATCC30234)* | Bom Jesus creek,  Belo Horizonte,  MG, Brazil | S 19°86084'  W 43° 99727' | 2017 |
| Farm Sewage |  |  |  |  |  |  |  |  |  | |  |  |  |  |  |  |  |
| 18.1 | Sewage | 3° | neg | neg | neg | pos | neg | neg | ND | | mimivirus-like | ND | ND | *A. castellanii (ATCC30234)* | Itaúna, MG,  Brasil | S 20°4'56.2044" | 2017 |
| 18.4 | Sewage | 3° | neg | neg | neg | pos | neg | neg | ND | | mimivirus-like | ND | ND | *A. castellanii (ATCC30234)* | Itaúna, MG,  Brasil | S 20°4'56.2044" | 2017 |
| 18.5 | Sewage | 2° | neg | ND | ND | neg | neg | neg | pos | | cedratvirus-like | ND | cedratvirus-like | *A. castellanii (ATCC30234)* | Itaúna, MG,  Brasil | S 20°4'56.2044" | 2017 |
| 18.10 | Sewage | 3° | pos | ND | ND | pos | neg | neg | ND | |  | ND | ND | *A. castellanii (ATCC30234)* | Itaúna, MG,  Brasil | S 20°4'56.2044" | 2017 |
| Water  treatment  station |  |  |  |  |  |  |  |  |  | |  |  |  |  |  |  |  |
| 19.1 | water | 3° | neg | neg | neg | pos | neg | neg | ND | | ND | ND | ND | *A. castellanii (ATCC30234)* | COPASA,  Belo Horizonte,  MG, Brazil | S 9°58'36.0876"  W 3°56'529368" | 2017 |
| 19.2 | water | 3° | neg | neg | neg | pos | neg | neg | ND | | ND | ND | ND | *A. castellanii (ATCC30234)* | COPASA,  Belo Horizonte,  MG, Brazil | S 19°58'36.0876"  W 43°56'529368" | 2017 |
| 19.4 | water | 3° | neg | neg | neg | pos | neg | neg | ND | | mimivirus-like | ND | ND | *A. castellanii (ATCC30234)* | COPASA,  Belo Horizonte,  MG, Brazil | S 19°58'36.0876"  W 43°56'529368" | 2017 |
| 19.5 | water | 3° | pos | ND | ND | pos | neg | neg | ND | | ND | ND | ND | *A. castellanii (ATCC30234)* | COPASA,  Belo Horizonte,  MG, Brazil | S 19°58'36.0876"  W 43°56'529368" | 2017 |
| 19.6 | water | 2° | neg | neg | neg | neg | neg | neg | ND | | ND | ND | mimivirus-like | *A. castellanii (ATCC30234)* | COPASA,  Belo Horizonte,  MG, Brazil | S 19°58'36.0876"  W 43°56'529368" | 2017 |
| 19.7 | water | 3° | neg | neg | neg | pos | neg | neg | ND | | mimivirus-like | ND | ND | *A. castellanii (ATCC30234)* | COPASA,  Belo Horizonte,  MG, Brazil | S 19°58'36.0876"  W 43°56'529368" | 2017 |
| 19.8 | water | 3° | neg | neg | neg | pos | neg | neg | ND | | mimivirus-like | ND | ND | *A. castellanii (ATCC30234)* | COPASA,  Belo Horizonte,  MG, Brazil | S 19°58'36.0876"  W 43°56'529368" | 2017 |
| 19.9 | water | 3° | pos | ND | ND | pos | neg | neg | ND | | ND | ND | ND | *A. castellanii (ATCC30234)* | COPASA,  Belo Horizonte,  MG, Brazil | S 19°58'36.0876"  W 43°56'529368" | 2017 |
| 19.10 | water | 3° | neg | neg | neg | pos | neg | neg | ND | |  | ND | ND | *A. castellanii (ATCC30234)* | COPASA,  Belo Horizonte,  MG, Brazil | S 19°58'36.0876"  W 43°56'529368" | 2017 |
| Antarctic |  |  |  |  |  |  |  |  |  | |  |  |  |  |  |  |  |
| PP | water | 3° | pos | ND | ND | pos | neg | neg | ND | | ND | ND | ND | *A. castellanii (ATCC30234)* | Punta Plaza,  Antarctica | S 62° 06. 169'  W 58° 21.375' | 2014 |
| PU | water | 3° | pos | ND | ND | pos | neg | neg | ND | | ND | ND | ND | *A. castellanii (ATCC30234)* | PuntaUllman,  Antarctica | S 62° 05. 090'  W 58° 20.592' | 2014 |
| YP | water | 3° | pos | ND | ND | pos | neg | neg | ND | | ND | ND | ND | *A. castellanii (ATCC30234)* | Yellow Point,  Antarctica | S 62° 05. 090'  W 58° 20.592' | 2014 |
| ART | water | 2° | pos | ND | ND | pos | neg | neg | ND | | ND | mimivirus-like | ND | *A. castellanii (ATCC30234)* | Arctowski,  Antarctica | S 62° 09. 246'  W 58° 27.207' | 2014 |
| Others |  |  |  |  |  |  |  |  |  | |  |  |  |  |  |  |  |
| CAP10 | Feces  Of  Capybara | 1° | pos | ND | ND | pos | neg | neg | ND | | ND | ND | ND | *A. castellanii (ATCC30234)* | Serra do Cipó,  MG,Brazil | S 19°20.457'  W 43°37.008' | 2012 |
| MG  collection | soil | 3° | neg | ND | ND | neg | neg | neg | ND | | mimivirus-like | ND | ND | *A. castellanii (ATCC30234)* | MG, Brazil | S 21° 64.617'  W 45°44.003' | 2014 |
| MG  collection | soil | 1° | neg | ND | ND | neg | neg | neg | ND | | ND | mimivirus-like | ND | *A. castellanii (ATCC30234)* | MG, Brazil | S 21° 23.437'  W 42°80.067' | 2014 |
| Pantanal | soil | 1° | neg | ND | ND | neg | neg | neg | ND | | ND | Unusual  morphology | Unusual  morphology | *A. castellanii (ATCC30234)* | Pantanal,  MT, Brazil | - | 2015 |
